# Congenital Pulmonary Airway Malformation Presenting as Pneumothorax: Two Case Reports

**DOI:** 10.1155/crpe/5078477

**Published:** 2026-08-27

**Authors:** Linda Paul Athman, Happiness Malyas, Joseph Nkuba, Aika Shoo

**Affiliations:** ^1^ Department of Paediatrics and Child Health, Kitete Regional Referral Hospital, Tabora, Tanzania; ^2^ Department of Paediatrics and Child Health, Muhimbili University of Health and Allied Sciences, Dar es Salaam, Tanzania, muchs.ac.tz; ^3^ Department of Paediatrics and Child Health, Muhimbili National Hospital, Dar es Salaam, Tanzania, mnh.or.tz

**Keywords:** congenital pulmonary airway malformation (CPAM), pneumothorax, respiratory distress

## Abstract

**Background:**

Congenital pulmonary airway malformation (CPAM) is a rare developmental anomaly of the lower respiratory tract, with an estimated incidence ranging from 1 in 10,000 to 1 in 35,000 live births. CPAM may present during infancy with severe respiratory distress and complications such as pneumothorax or recurrent pulmonary infection.

**Case Presentation:**

We report two female infants aged 7 months and 2 months who presented with severe respiratory distress and radiological findings initially suggestive of pneumothorax. In Case 1, chest computed tomography (CT) demonstrated a large solitary cyst consistent with Type I CPAM. The patient required prolonged pediatric intensive care admission complicated by ventilator‐associated infection with *Pseudomonas aeruginosa* and *Acinetobacter baumannii* before successful right lower lobectomy and recovery. In Case 2, chest CT demonstrated multiple cystic hyperinflated lesions suggested Type III CPAM; however, alternative differentials of bronchial atresia and other congenital cystic lung lesions could not be excluded. Despite aggressive ventilatory and antimicrobial management, the infant deteriorated and died from progressive respiratory failure prior to surgical intervention.

**Conclusion:**

These cases highlight the diagnostic complexity of congenital cystic lung lesions presenting with respiratory distress in resource‐limited settings. Early advanced imaging, multidisciplinary evaluation, and timely surgical management remain critical for improving outcomes.

## 1. Introduction

Congenital pulmonary airway malformation (CPAM), formerly known as congenital cystic adenomatoid malformation is a rare developmental disorder of the lower respiratory tract [1]. The incidence ranges from approximately 1 in 10,000 to 1 in 35,000 live births. Although rare, it the most common type of lung malformation. The lesion results from an embryologic injury during early fetal life, with maldevelopment of terminal bronchiolar structures during the 4th–7th week of gestation [2]. CPAM is classified into 5 types, from Type 0 to Type IV, depending upon the origin of pulmonary areas of the lung, cyst size, and cyst appearance [2]. Clinical presentation of CPAM is variable ranging from asymptomatic to patients with respiratory distress, recurrent chest infections, and pneumothorax. Diagnosis is made prenatally by using an ultrasound; however, few cases are diagnosed during childhood or even adulthood. Treatment of CPAM for symptomatic patients is surgical resection, with timing dependent on the severity of symptoms [3].

## 2. Case 1

A 7‐month‐old female presented with a 2‐month history of productive cough, intermittent episodes of low‐to‐high‐grade fever, and progressive difficulty in breathing. Upon admission, she was unable to breastfeed due to severe respiratory distress, which resulted in significant weight loss. There was no known history of tuberculosis (TB) contact. Pregnancy and birth history were unremarkable. She was the third‐born in the family, and her siblings were reportedly healthy.

Upon examination, the patient was in severe respiratory distress, with oxygen saturation of < 92% on room air, head nodding, and severe lower chest wall indrawing. She was febrile (*T* = 38.8°C) and severely pale, with no palpable lymphadenopathy. Chest auscultation revealed reduced breath sounds on the right side, while other systemic examinations were unremarkable.

Initially, she was managed as a case of severe pneumonia with a right‐sided pneumothorax. A chest x‐ray revealed a mediastinal shift with well‐demarcated radiolucent areas, suggestive of tension pneumothorax secondary to severe staphylococcal pneumonia. Urgent needle decompression was performed, followed by chest tube insertion. She was started on intravenous dexamethasone, meropenem, and vancomycin and subsequently transferred to the pediatric intensive care unit (PICU) due to persistent respiratory distress despite thoracostomy.

In the PICU, she was intubated and mechanically ventilated for over 3 weeks. During that time, she developed ventilator associated infection as tracheal aspirate cultures grew *Pseudomonas aeruginosa* and *Acinetobacter baumannii* necessitating addition of a targeted antibiotic therapy, amikacin for 7 days based on sensitivity testing. Multiple attempts at extubation were unsuccessful due to recurrent respiratory distress after extubation.

Follow‐up chest x‐rays showed an accumulation of air in the right lower lobe, suggesting a pneumatocele with resolved pneumothorax. A chest computed tomography (CT) scan confirmed the presence of a solitary large air‐filled cyst with consolidative changes.

Given the findings, consistent with CPAM Type I, surgery was scheduled and the patient underwent a right lower lobectomy with cyst resection and biopsy. Intraoperative findings revealed a large cystic lesion occupying the right lower quadrant, with adjacent smaller cystic lesions communicating with the sequential bronchus. Complete anatomical dissection and resection of the right lower lobe were performed successfully.

The child was managed in the PICU postoperatively before being transferred to the pediatric ward. Follow‐up chest x‐rays showed lung re‐expansion initially but later demonstrated right lung collapse with tracheal deviation toward the affected side. Clinically, she had residual respiratory distress with stridor and was managed with continuous nebulization and chest physiotherapy. Subglottic stenosis was ruled out, and gradual improvement was observed. A month later, she was weaned off oxygen and discharged home. The parents were counseled on continued care and scheduled for follow‐up visits.

## 3. Case 2

A 2‐month‐old female infant was admitted with a 2‐week history of recurrent fever and productive cough, which progressed to difficulty in breathing 5 days prior to admission. There was no known TB contact or similar symptoms among her siblings. Pregnancy and birth history were unremarkable. She had received all vaccinations as per age and was exclusively breastfed with adequate weight gain. At 2 months of age, she had a social smile and adequate neck control.

On admission, she was on oxygen therapy due to severe respiratory distress, with a respiratory rate of > 60 breaths per minute, peripheral and central cyanosis, lower chest wall indrawing, and intercostal recession. She was afebrile (*T* = 37.4°C) and had no palmar pallor. Chest examination revealed asymmetrical chest expansion, more pronounced on the left side, with hyper‐resonance and reduced breath sounds. Cardiovascular and other systemic examinations were unremarkable.

A diagnosis of severe pneumonia with right‐sided spontaneous pneumothorax was made. A chest x‐ray revealed a hyperlucent left lung with tracheal and mediastinal shift to the left. Immediate needle thoracocentesis was performed, releasing a gush of air, and a chest tube was subsequently inserted. She was started on intravenous vancomycin and ceftriaxone–sulbactam, targeting *Staphylococcus* pneumonia, which was later confirmed by blood culture results showing *Staphylococcus* species sensitive to cefoxitin and clindamycin. The blood culture isolate was not speciated beyond Staphylococcus species, which represents a limitation of the available microbiological workup.

After 48 hours of chest tube insertion, follow‐up x‐rays showed no significant improvement. Her respiratory distress worsened, necessitating intubation and mechanical ventilation. A chest CT scan revealed regional hyperinflation of the right lung, composed of multiple cysts of varying sizes, leading to relaxation collapse of the anterior segment of the upper lobe with mediastinal shift to the contralateral side. These findings were suggestive of CPAM Type III; however, bronchial atresia and other congenital cystic lung lesions remained differential considerations.

Bronchoscopy prior to surgery showed a normal tracheobronchial tree but revealed right bronchial compression and white patches on the tracheal wall. Ventilator‐associated pneumonia with high suspicion of fungal infection based on bronchoscopy findings, and the treatment regimen was adjusted to include intravenous fluconazole (120‐mg loading dose and then 60 mg once daily), piperacillin–tazobactam (490‐mg TDS), and amikacin (90‐mg OD). Despite aggressive medical and supportive management, the infant’s condition deteriorated. She succumbed to respiratory failure a week later and passed away.

## 4. Discussion

CPAM, formerly known as congenital cystic adenomatoid malformation, is a rare developmental anomaly of the lower respiratory tract. It occurs in approximately 1 in 10,000 to 1 in 35,000 live births and represents the most common type of congenital lung malformation. CPAM results from abnormal embryologic development of the terminal bronchiolar structures, leading to cystic lesions in the lung [1, 2].

CPAM can be classified into five types based on the origin of the affected pulmonary structures, cyst size, and histological characteristics [3]. Type 0: Rare and often lethal, involving the trachea and bronchi, with small cystic or solid lung lesions. Type I: The most common form, characterized by large cysts (> 2 cm) lined with ciliated pseudostratified epithelium. Type II: Composed of smaller cysts (< 2 cm) and associated with other congenital anomalies. Type III: Solid, microcystic lung lesions involving the entire lobe, often leading to respiratory distress. Type IV: Large peripheral cystic lesions that may resemble pleuropulmonary blastoma.

The diagnosis of CPAM requires a combination of clinical, radiological, and histological findings. Clinically, CPAM presents variably, from asymptomatic cases detected incidentally to severe respiratory distress, recurrent pneumonia, or pneumothorax. In resource‐limited settings, its nonspecific symptoms often lead to misdiagnosis or delayed diagnosis, as CPAM can mimic severe pneumonia or TB, especially when advanced imaging like CT chest is unavailable [4].

In Africa and other low‐resource settings, diagnosing and managing CPAM is particularly challenging due to limited access to radiological imaging, including CT scans, which are essential for confirming the diagnosis. Most cases are treated empirically as pneumonia due to the lack of specialized diagnostic tools. A severe shortage of pediatric cardiothoracic surgeons, for example, in Tanzania, only two cardiothoracic surgeons are available at the national referral hospital, leading to delays in definitive surgical treatment.

**FIGURE 1:**
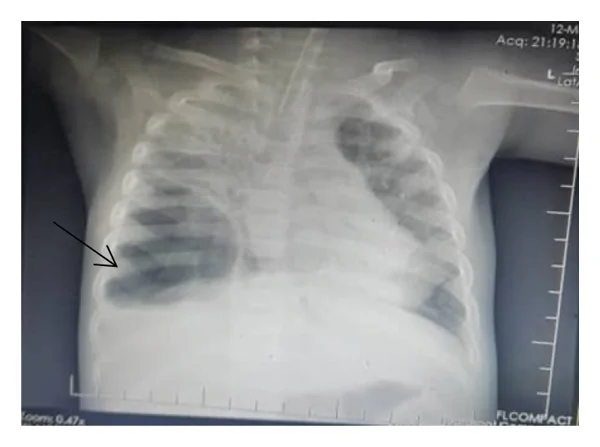
Chest x‐ray revealed large air‐filled cysts on the right hemithorax, with consolidation on the left lung and homogenous opacification of the right upper and middle lobe.

**FIGURE 2:**
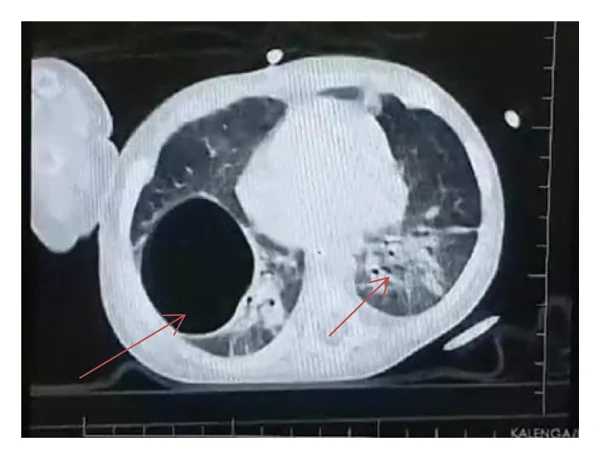
Axial view of CT scan of the chest showing a solitary large air‐filled cyst with consolidative changes in the lung fields.

**FIGURE 3:**
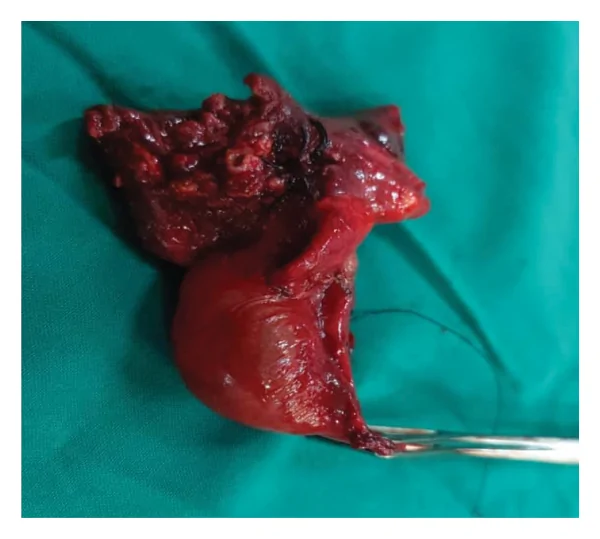
Lower segment of the right lung with a cyst that was resected.

**FIGURE 4:**
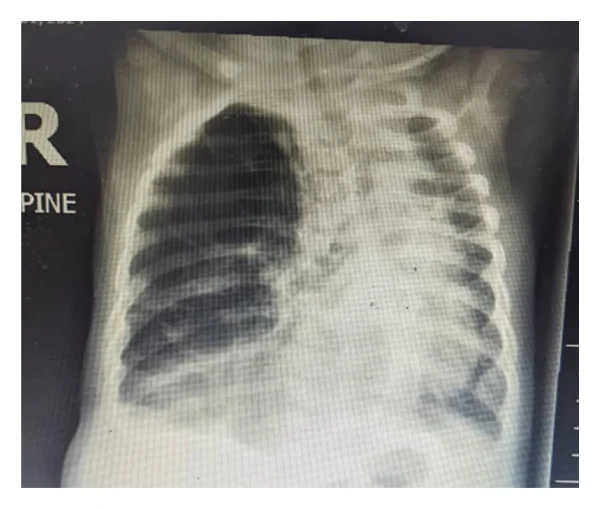
Chest x‐ray image taken (on admission) revealed hyperlucense on left hemithorax with trachea and mediastinal shift to the left.

**FIGURE 5:**
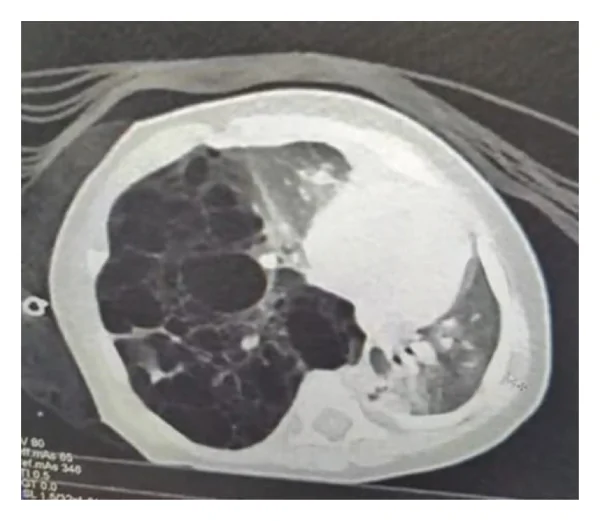
CT scan chest with contrast (axial view) shows regional hyperinflation on the right, composed of cysts of different sizes causing relaxation collapse of the anterior segment of the upper lobe with mediastinal shift to the contralateral side.

In the first case, a 7‐month‐old infant presented with cough, fever, and respiratory distress for 1 month. Despite initial management with needle decompression and chest tube insertion, the condition worsened, necessitating PICU admission. A CT scan confirmed CPAM, and the patient underwent a right lobectomy with cyst resection, leading to a successful recovery (Figures 1, 2, 3, 4, and 5).

In contrast, the second case involved a 2‐month‐old infant with recurrent fever and respiratory distress. Despite aggressive treatment, including antimicrobial therapy and mechanical ventilation, the infant deteriorated before surgery could be performed. The unfavorable outcome in Case 2 was likely multifactorial. Contributing factors included severe respiratory compromise at presentation, delayed definitive surgical intervention, progressive ventilator dependence, suspected ventilator‐associated infection, and persistent mass effect from the underlying congenital lesion. In addition, prolonged critical illness may have contributed to inflammatory lung injury and impaired pulmonary reserve. These findings underscore the importance of early referral and multidisciplinary surgical evaluation in infants with persistent respiratory deterioration despite adequate drainage and antimicrobial therapy.

The radiological interpretation of Case 2 remained diagnostically challenging. Although the imaging findings were initially interpreted as Type III CPAM, alternative congenital cystic lung lesions, including bronchial atresia with secondary hyperinflation, could not be fully excluded. The marked unilateral hyperinflation and mediastinal shift observed on imaging may overlap with several congenital pulmonary anomalies, particularly in critically ill infants. Histopathological confirmation was not obtained because the patient deteriorated before definitive surgical intervention could be performed. This limitation highlights the diagnostic challenges encountered in resource‐limited settings where access to subspecialized pediatric thoracic radiology and advanced multidisciplinary evaluation may be limited.

The systematic review from the APSA committee mentions that observations strategies for asymptomatic CPAMs include at least one postnatal CT scan and the timing of scan depends on practitioner’s management strategy. The article also mentions “optimal timing” for resection—immediate postnatal resection is not mandatory for the asymptomatic patient. While there is little evidence to mandate an operation at a defined time in the first year of life, the current available data would suggest that early (< 6 months of age) surgery allows for ease of operative intervention, adequate recovery, and a reasonable time for compensatory lung growth, while avoiding the potential infectious complications associated with observation [5].

These cases highlight the importance of early recognition of CPAM in infants with unexplained respiratory distress and pneumothorax. Early imaging, including chest x‐ray and CT scan, is crucial for accurate diagnosis. In severely symptomatic cases, timely surgical intervention is essential for survival. A multidisciplinary approach involving pediatric intensivists, pulmonologists, and surgeons is vital to improving outcomes, particularly in resource‐limited settings where specialized care is scarce [6, 7].

## 5. Conclusion

CPAM is a rare but significant cause of respiratory distress in infants. Early diagnosis and prompt surgical intervention are critical for favorable outcomes. The cases presented highlight the importance of timely recognition and treatment, the need for aggressive management of secondary infections, and the challenges faced in managing critically ill infants with CPAM.

**Nomenclature**
CPAM: Congenital pulmonary airway malformation
CT: Computed tomography
PICU: Pediatric intensive care unit

## Funding

No funding was received for this manuscript.

## Ethics Statement

Written informed consent was obtained from the patient’s legal guardians for publication of this case report and any accompanying images.

## Conflicts of Interest

The authors declare no conflicts of interests.

## Supporting Information

Additional supporting information can be found online in the Supporting Information section.

## Supporting information


**Supporting Information** Completed Care Checklist.

## Data Availability

Data used to support the findings of this study are available from the corresponding authors upon reasonable request.
