# Supplementary material for: Congenital Pulmonary Airway Malformation Presenting as Pneumothorax: Two Case Reports
Source: Case Rep Pediatr. 2026 Aug 27;2026:5078477. doi: 10.1155/crpe/5078477 (PMC13522626; doi:10.1155/crpe/5078477)
Supplement: Supplementary file 1 [file CRPE-2026-5078477-s001.docx]

COMPLETED CARE CHECKLIST

| **CARE Item** | **Description** | **Reported in Manuscript** |
| --- | --- | --- |
| **1. Title** | Includes "case report" and the diagnosis | **Yes.** The title identifies CPAM and states "Two Case Reports." |
| **2. Key words** | 2–5 key words | **Yes.** CPAM, pneumothorax, respiratory distress. |
| **3a. Abstract – Introduction** | What is unique about the case? | **Yes.** Rare presentation of CPAM presenting as pneumothorax. |
| **3b. Abstract – Case presentation** | Main symptoms, diagnosis, intervention, outcome | **Yes.** Both cases summarized. |
| **3c. Abstract – Conclusion** | Main lessons | **Yes.** Early imaging and multidisciplinary management emphasized. |
| **4. Introduction** | Background and rationale | **Yes.** CPAM epidemiology, classification and management described. |
| **5a. Patient information** | Demographics | **Yes.** Age and sex reported for both infants. |
| **5b. Main symptoms** | Presenting complaints | **Yes.** Respiratory distress, cough, fever. |
| **5c. Medical, family and psychosocial history** | Relevant history | **Partially.** Pregnancy, birth and family history are included, but psychosocial history is not applicable for infants. |
| **6. Clinical findings** | Physical examination | **Yes.** Comprehensive examination findings are reported. |
| **7. Timeline** | Timeline of events | **No.** The manuscript presents events chronologically but does **not include a timeline figure or table.** |
| **8a. Diagnostic assessment** | Diagnostic methods | **Yes.** Chest X-ray, CT scan, bronchoscopy, cultures. |
| **8b. Diagnostic challenges** | Financial, access or diagnostic limitations | **Yes.** Resource limitations and diagnostic uncertainty are discussed. |
| **8c. Diagnostic reasoning** | Differential diagnoses | **Yes.** Bronchial atresia and other congenital cystic lung lesions considered. |
| **8d. Prognostic characteristics** | Where applicable | **Not applicable.** |
| **9a. Therapeutic intervention** | Type of intervention | **Yes.** Antibiotics, ventilation, thoracostomy, lobectomy. |
| **9b. Administration details** | Dose, duration, procedures | **Yes.** Drug regimens and surgery are described. |
| **9c. Changes in intervention** | Modifications with rationale | **Yes.** Antibiotics changed according to culture results and clinical response. |
| **10a. Follow-up and outcomes** | Clinical course | **Yes.** Recovery in Case 1 and death in Case 2 documented. |
| **10b. Follow-up tests** | Laboratory/imaging | **Yes.** Serial imaging and cultures reported. |
| **10c. Intervention adherence** | Adherence | **Not applicable.** |
| **10d. Adverse events** | Unexpected events | **Yes.** Ventilator-associated infection and postoperative complications reported. |
| **11. Discussion** | Strengths, limitations, literature comparison, lessons | **Yes.** Comprehensive discussion with literature comparison and clinical implications. |
| **12. Patient perspective** | Patient/family perspective | **No. Missing.** |
| **13. Informed consent** | Written consent obtained | **Yes.** Guardian consent stated. |
